# Unfolding grain size effects in barium titanate ferroelectric ceramics

**DOI:** 10.1038/srep09953

**Published:** 2015-05-07

**Authors:** Yongqiang Tan, Jialiang Zhang, Yanqing Wu, Chunlei Wang, Vladimir Koval, Baogui Shi, Haitao Ye, Ruth McKinnon, Giuseppe Viola, Haixue Yan

**Affiliations:** 1 School of physics, State Key Laboratory of Crystal Materials, Shandong University, Jinan 250100, P. R. China; 2 Institute of Materials Research, Slovak Academy of Sciences, Watsonova 47, 040 01 Kosice, Slovakia; 3 School of Engineering and Applied Science, Aston University, Birmingham B4 7ET, United Kingdom; 4 School of Engineering and Materials Science, Queen Mary University of London, Mile End Road, London, E1 4NS, United Kingdom; 5 Department of Applied Science and Technology, Institute of Materials Physics and Engineering, Corso Duca degli Abruzzi 24, 10129 Torino, Italy

## Abstract

Grain size effects on the physical properties of polycrystalline ferroelectrics have been extensively studied for decades; however there are still major controversies regarding the dependence of the piezoelectric and ferroelectric properties on the grain size. Dense BaTiO_3_ ceramics with different grain sizes were fabricated by either conventional sintering or spark plasma sintering using micro- and nano-sized powders. The results show that the grain size effect on the dielectric permittivity is nearly independent of the sintering method and starting powder used. A peak in the permittivity is observed in all the ceramics with a grain size near 1 μm and can be attributed to a maximum domain wall density and mobility. The piezoelectric coefficient *d*_33_ and remnant polarization *P*_r_ show diverse grain size effects depending on the particle size of the starting powder and sintering temperature. This suggests that besides domain wall density, other factors such as back fields and point defects, which influence the domain wall mobility, could be responsible for the different grain size dependence observed in the dielectric and piezoelectric/ferroelectric properties. In cases where point defects are not the dominant contributor, the piezoelectric constant *d*_33_ and the remnant polarization *P*_r_ increase with increasing grain size.

Understanding the grain size effects that govern crystal structure and the functional properties of ferroelectrics is of vital importance in improving the performance of ferroelectric systems, which are embedded in a number of electronic devices, such as sensors, actuators, transducers and non-volatile memories^1^,^2^,^3^. Due to a growing demand for miniature devices, significant progress in the fabrication of micro-, meso- and nano-scale ferroelectric structures has been made^4^,^5^. A fundamental understanding of grain size effects on the dielectric and ferroelectric properties was achieved by studying low-dimensional ferroelectric structures^6^,^7^,^8^,^9^,^10^,^11^,^12^. Theoretical and experimental studies on thin/ultrathin films^6^,^7^,^8^, nanowires^9^,^10^ and other types of nano-dimensional systems^11^,^12^ have shown that ferroelectricity persists down to the nanoscale, thereby demonstrating their potential for use in miniature devices. Nevertheless, certain applications require bulk components with specific functional properties, which can be directly obtained from a specific grain size. Although grain size effects on the dielectric, piezoelectric and ferroelectric properties have been widely studied in several ferroelectric bulk systems, there are still a number of aspects which remain unclear. These are mainly related to the grain size dependency of the piezoelectric and ferroelectric properties, often showing discrepancies in the existing literature. In addition, there are a number of other factors that could influence the grain size dependency; their identification is the main aim of the present study. Barium titanate ceramics are chosen as a model ferroelectric system for this research.

Barium titanate (BaTiO_3_) is a typical ferroelectric material with a perovskite-type structure. It has been widely studied for dielectric capacitor and lead-free piezoelectric applications, achieving both high dielectric permittivity (up to 7000)^13^ and piezoelectric constant (*d*_33_ up to 788 pC/N in textured ceramics)^14^ values. In BaTiO_3_ ceramics, the grain size has been reported to have substantial influence on the dielectric permittivity^13^,^15^,^16^,^17^,^18^,^19^,^20^,^21^. The dielectric constant of the BaTiO_3_ ceramics first increases with decreasing average grain size, reaching a maximum value in the ~ 0.8–1.1 μm grain size range, and then rapidly decreases with further decrease of grain size^13^,^15^,^16^,^17^,^18^,^19^,^20^,^21^. Similar behaviour has been observed in other ferroelectrics^22^,^23^,^24^. Generally, the grain size dependence of the dielectric permittivity shows consistent trends despite the use of different powder processing and sintering methods^13^,^15^,^16^,^17^,^18^,^19^,^20^,^21^. Regarding the physical origin of the maximum value of the permittivity, usually associated with an intermediate grain size of ~ 1 μm, two alternate theories based on internal residual stress and domain wall motion have been developed over the past decades (see Ref. 19 for a review). Recent in situ high energy X-ray diffraction experiments performed on BaTiO_3_ ceramics with grain sizes in the range 0.32–3.5 µm suggest that the maximum permittivity found around 1 µm grain size is due to maximum domain wall contribution^21^.

On the other hand, the reported piezoelectric coefficient *d*_33_ and its grain size dependence have shown contradicting results in the literature^14^,^21^,^25^,^26^,^27^,^28^,^29^,^30^,^31^,^32^. For several decades, it has been accepted that the BaTiO_3_-based ceramics show only modest piezoelectric activity, with a piezoelectric constant *d*_33_ lower than 190 pC/N^25^. Nevertheless, remarkably high *d*_33_ values (up to 460 pC/N) have been obtained over recent years for BaTiO_3_ ceramics (grain size around 1 μm) prepared from hydrothermally synthesized fine powders^14^,^26^,^27^. In addition, a peak of 338 pC/N in the piezoelectric constant was found around the grain size of 1 μm in BaTiO_3_ ceramics prepared by solid-state reaction and conventional sintering^28^. Using conventional sintering, it is difficult to obtain dense BaTiO_3_ ceramics with an average grain size smaller than 1 μm, the relatively low density of these ceramics is considered to have caused the reduction of the piezoelectric constant when compared to the ceramics prepared from hydrothermal powders^28^. The BaTiO_3_ ceramics fabricated by the conventional solid state reaction route with a bimodal grain size distribution (large grains had an average size of about 7.0 µm and small grains had an average size of about 0.8 μm) showed a high *d*_33_ value of 419 pC/N, which could not be solely attributed to a grain size effect^29^. Recently, a peak in the *d*_33_ was found around 2 µm grain size in barium titanate ceramics and it was concluded that like the permittivity, the maximization of the piezoelectric constant *d*_33_ is due to movement of 90° domain walls^21^. However, for BaTiO_3_ ceramics prepared from ultrafine powders with grain size ranging from 0.56 to 120 μm, a maximum *d*_33_ value was reported at 8.9 μm^30^, which is larger than the grain size reported in other studies (1–2 μm)^21^. Additional discrepancies in the grain size dependence of the piezoelectric constant can be found in Refs. 31, 32.

These differences demonstrate that processing conditions can have a significant influence on the physical properties of ferroelectric ceramics. However, at present, the detailed mechanisms for the grain size dependence of the piezoelectric properties in the BaTiO_3_ ceramics prepared using different processing methods and different starting materials are still unclear.

To solve this controversy, the dependence of the dielectric, piezoelectric and ferroelectric properties on the average grain size of barium titanate ceramics prepared by conventional sintering (CS) and spark plasma sintering (SPS) using micro- and nano-sized powders (see Fig. S1 in supplementary figures) was systematically studied. SPS is an effective way to fabricate dense nanostructured ceramics^21^,^23^,^33^, which could allow for the preparation of a series of fully dense BaTiO_3_ ceramics with a large range of grain sizes. Through this systematic comparison, the present study highlights the main factors responsible for the controversial grain size dependence previously reported. In addition, it also provides an advanced understanding of the underlying mechanisms of the grain size effect, which will have important implications in maximizing the dielectric, piezoelectric and ferroelectric/ferroelastic properties of other ferroelectric systems.

## Results

### Grain size dependence of permittivity and piezoelectric constant

Table 1 summarizes the room temperature dielectric and piezoelectric properties of poled BaTiO_3_ ceramics prepared by conventional sintering and SPS from the micro- and nano-sized BaTiO_3_ powders. In the table, the labels ‘CS’ and ‘SPS’ denote ‘conventional sintering’ and ‘spark plasma sintering’, respectively; the labels ‘micro’ and ‘nano’ describe ceramics sintered using micro- and nano-sized BaTiO_3_ powder, respectively; while the number after them indicates the sintering temperature. The values of the relative density, average grain size, piezoelectric constant *d*_33_, orthorhombic-tetragonal phase transition temperature *T*_O-T_ and Curie point *T*_C_ are also listed in the table. The *T*_O-T_ and *T*_C_ values were determined from the peaks in the permittivity vs. temperature curves measured during heating. From Table 1 it can be seen that all BaTiO_3_ samples show high density. For CS-micro BaTiO_3_ ceramics, the relative density gradually increases from 95.2% to 98.6% and the average grain size increases from 1.3 μm to 32.0 μm with increasing sintering temperature. The relative density of all the SPS samples is larger than 98.5% and the average grain size increases from 0.6 μm to 18.5 μm with increasing sintering temperature.

**Figure 1:**
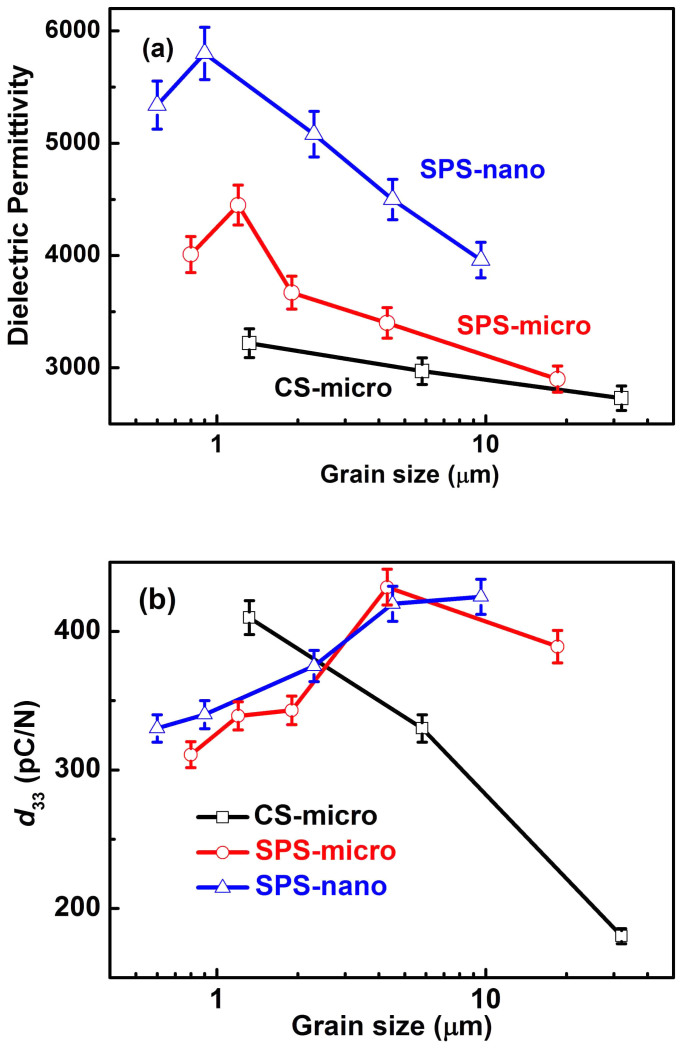
Grain size dependence of (a) the dielectric permittivity, and (b) the piezoelectric *d*_33_ constant of the poled BaTiO_3_ ceramics prepared by the conventional and SPS techniques from micro- and nano-sized powders. The conventional sintering temperatures are 1230°C, 1280°C and 1350°C; the SPS sintering temperatures for the micro-sized powder are 1080°C, 1100°C, 1120°C, 1200°C and 1240°C, and for the nano-sized powder are 1000°C, 1020°C, 1040°C, 1060°C and 1160°C.

The room temperature grain size dependence of the permittivity and piezoelectric constant of the poled BaTiO_3_ ceramics prepared by CS and SPS techniques are plotted using the data in Table 1 and the results are shown in Fig. 1a and Fig. 1b. The dielectric permittivity *ε*' shows a similar grain size dependence in ceramics sintered by the CS and SPS, as shown in Fig. 1a. The *ε*' value of the CS-micro ceramics increases from 2730 to 3220 with decreasing grain size (Fig. 1a). Analogously to CS, the permittivity of the SPS ceramics increases with the reduction of grain size and shows a peak around 1 μm. After further reduction of the grain size, the permittivity decreases (Fig. 1a). For SPS-micro BaTiO_3_ ceramics, the ceramic with an average grain size of 1.2 μm shows the largest permittivity of 4450. The permittivity of the SPS-nano ceramics shows a peak value of 5800 at around 1 μm grain size (Fig. 1a). It can be seen that SPS BaTiO_3_ ceramics exhibit a larger permittivity than that of the CS-micro ceramics in the whole grain size range. Furthermore, for SPS ceramics, SPS-nano ceramics exhibit a larger permittivity than SPS-micro ceramics in the whole grain size range (Fig. 1a). The grain size dependence of the permittivity observed in this study is in good agreement with the results reported in the literature, although the values show some variations due to the different raw powders and sintering techniques used^13^,^15^,^16^,^17^,^18^,^19^,^20^,^21^.

The piezoelectric constant *d*_33_ shows the opposite grain size dependence for the two different sintering methods (Fig. 1b). CS-micro BaTiO_3_ ceramics show a maximum *d*_33_ value of 410 pC/N in CS-micro-1230 ceramic with an average grain size of 1.3 μm, which remarkably decreases with increasing grain size (Fig. 1b). The ceramic sintered at 1350°C shows a *d*_33_ value of about 180 pC/N (Fig. 1b), which is consistent with the previous results for coarse grained BaTiO_3_ ceramics^25^,^28^. When using the SPS, the piezoelectric constant of the SPS-micro ceramics increases with increasing grain size and shows a maximum value of 430 pC/N in SPS-micro-1200 ceramic with an average grain size of about 4 μm (Fig. 1b). The *d*_33_ value decreases with further increase of the sintering temperature above 1200°C (Fig. 1b). Owing to the fine particle size, the sintering temperatures of the SPS-nano ceramics are much lower than those of ceramics prepared from the micro-sized powder. All the SPS BaTiO_3_ ceramics prepared from the nano-sized powder show a high *d*_33_ value (> 300 pC/N). The maximum *d*_33_ of SPS-nano BaTiO_3_ ceramics is close to the maximum *d*_33_ of the SPS-micro ceramics. However, the *d*_33_ coefficient of the SPS-nano ceramics, unlike that of the SPS-micro ceramics, increases with increasing grain size up to 9.6 μm. By comparing the piezoelectric constant of ceramics sintered by the two different methods, it can be inferred that there is a critical grain size of about 2 μm below which CS-micro BaTiO_3_ ceramics show a larger *d*_33_. The maximum *d*_33_ for both the BaTiO_3_ ceramics sintered by the CS and SPS show a remarkably large value of over 400 pC/N.

### Grain size dependence of polarization-electric field hysteresis loops

**Figure 2:**
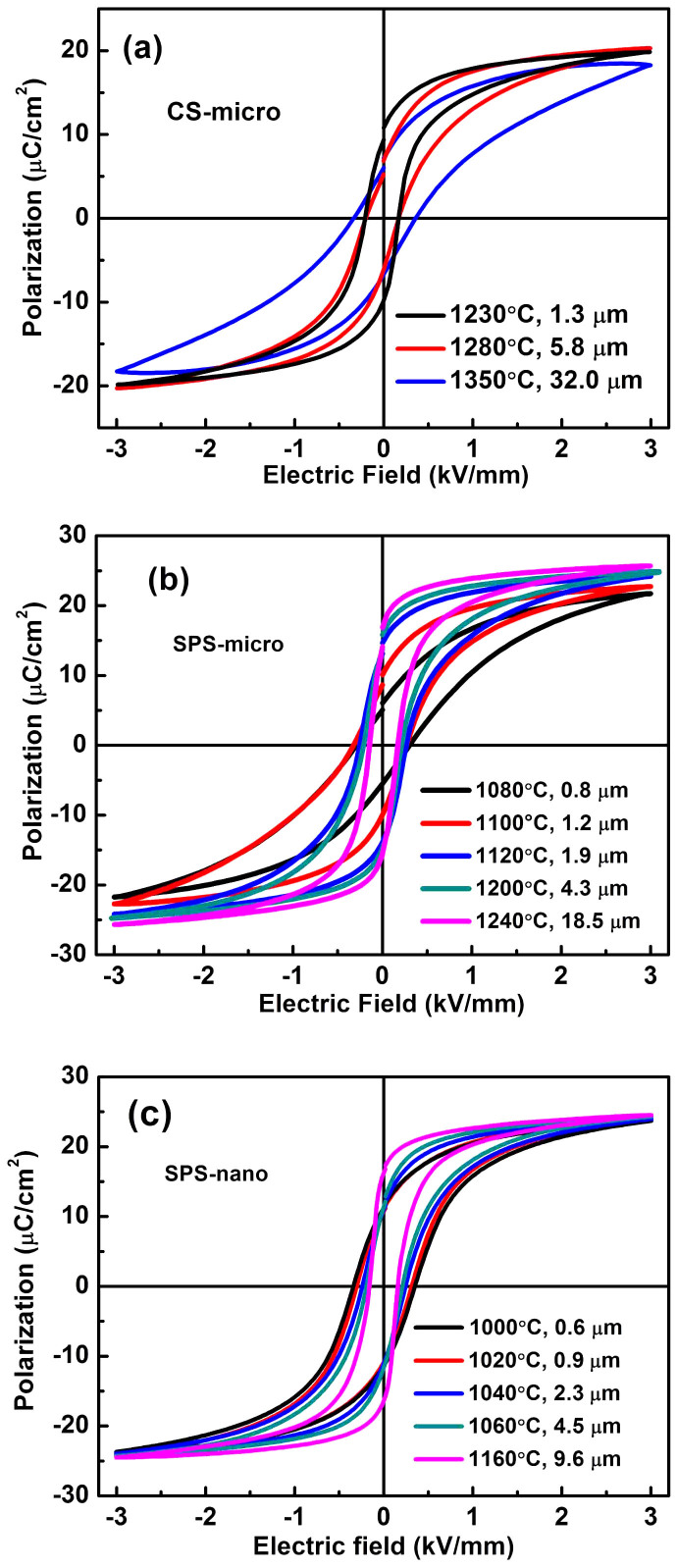
P-E loops of BaTiO_3_ ceramics prepared from different powders and sintered at different temperatures. (a) CS-micro ceramics; (b) SPS-micro ceramics; (c) SPS-nano ceramics.

**Figure 3:**
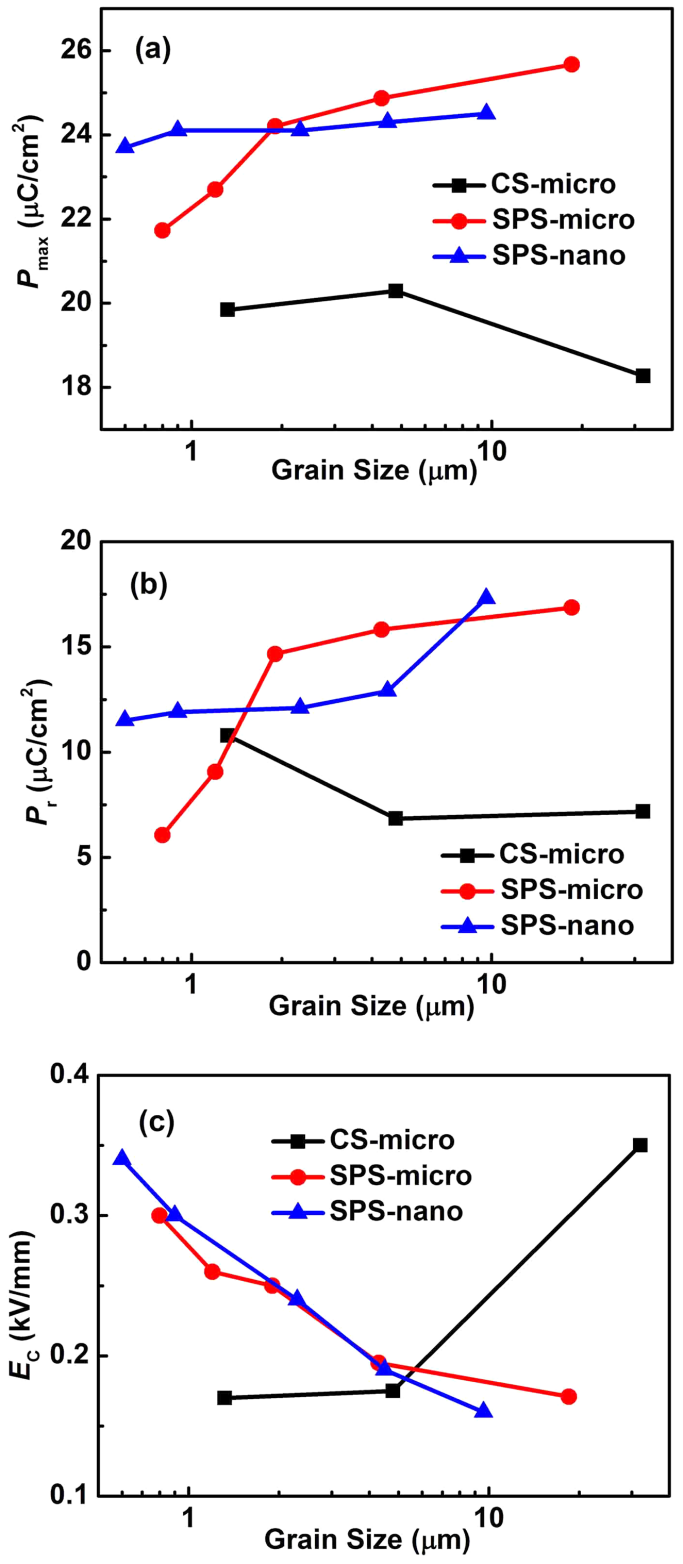
Grain size dependence of (a) maximum polarization *P*_max_, (b) remnant polarization *P*_r_ and (c) coercive field *E*_C_ for BaTiO_3_ ceramics prepared by different sintering methods from micro- and nano-sized powders.

Figure 2 shows the polarization-electric field (P-E) hysteresis loops of the unpoled BaTiO_3_ ceramics sintered by the CS and SPS. All of the ceramics except CS-micro-1350 show slim P-E hysteresis loops with a coercive field (*E*_c_) lower than 0.35 kV/mm, which is close to the value reported by other researchers^25^,^31^. The grain size dependence of the maximum polarization (*P*_max_) and remnant polarization (*P*_r_) of the CS and SPS BaTiO_3_ ceramics is shown in Fig. 3. It can be seen that for CS ceramics, both *P*_max_ and *P*_r_ decrease with increasing grain size (Figs. 3a, 3b). The CS-micro samples show a larger *P*_r_ than the SPS ceramics when the grain size is below 2 μm, and a lower value when the grain size is above 2 μm (Fig. 3b). In the SPS-micro ceramics the maximum polarization *P*_max_ slightly increases with increasing grain size and the values are larger than those of the CS-micro samples. The remnant polarization *P*_r_ slightly increases and gradually saturates for grain sizes larger than 2 µm; meanwhile it dramatically decreases when the grain size is below 2 μm. For the SPS-nano ceramics, the *P*_max_ slightly increases with increasing grain size and *P*_r_ dramatically increases for grain sizes larger than 4.5 µm (Fig. 3b), which is consistent with the grain size dependence of *d*_33_ shown in Fig. 1. Figure 3c shows that the CS-micro-1350 sample (32 µm average grain size) exhibits a coercive field of 0.35 kV/mm which is larger than that of the CS-micro-1230 (1.3 µm average grain size; *E*_c_ = 0.17 kV/mm) and CS-micro-1280 ceramics (5.8 µm average grain size; *E*_c_ = 0.175 kV/mm). For the SPS-micro ceramics, *E*_c_ gradually decreases from 0.3 kV/mm to 0.17 kV/mm with increasing grain size. A similar grain size dependence of the coercive field is shown by the SPS-nano ceramics (Fig. 3c).

### Grain size dependence of domain structure

**Figure 4:**
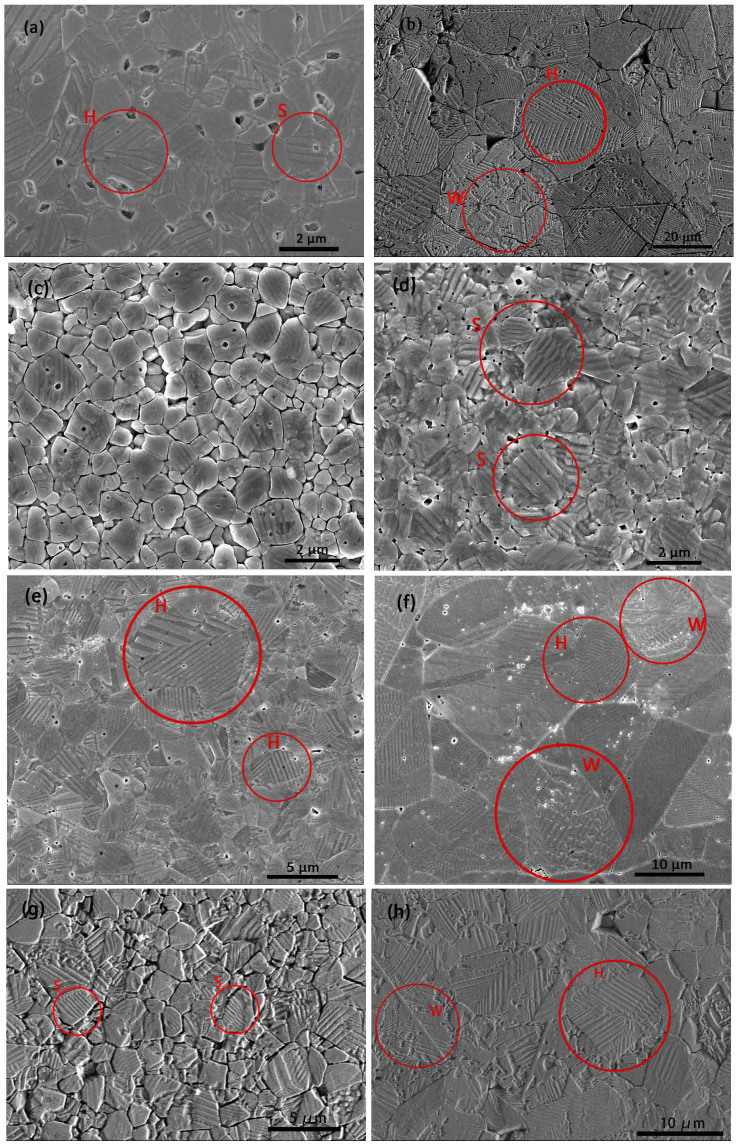
Domain structures of BaTiO_3_ ceramics. (a) CS-micro-1230; (b) CS-micro-1350; (c) SPS-micro-1080; (d) SPS-micro-1120; (e) SPS-micro-1200; (f) SPS-micro-1240; (g) SPS-nano-1040; (h) SPS-nano-1160. ‘S’ denotes stripe domain structure, ‘H’ denotes herringbone domain structure and ‘W’ denotes watermark domain structure.

Figure 4 shows SEM images of the domain patterns of several poled BaTiO_3_ ceramics prepared from micro- and nano-sized powders. The microstructures of the CS-micro ceramics are displayed in Figs. 4a and 4b. Figs. 4c to 4f show images of the SPS-micro ceramics, while Figs. 4g and 4h show the domain structures of two typical SPS-nano ceramics. For fine-grained BaTiO_3_ ceramics, the domain patterns mainly consist of stripes marked as ‘S’ running across the whole grain, as visible in Figs. 4a and 4c. The stripe lengths increase and the average domain width becomes larger as the grain size increases. The stripes are believed to correspond to 90° domain patterns^34^,^35^. The formation of 90° domains is a consequence of the relief of internal stresses in the BaTiO_3_ ceramics when cooled from a high temperature to below *T*_C_^34^,^35^. Herringbone patterns marked as ‘H’ consisting of two adjacent sets of parallel stripes were occasionally found, especially in coarse grained samples with grain sizes larger than 2 μm, as shown in Figs. 4b, 4e, 4f and 4h. These have been reported as typical domain structures of tetragonal BaTiO_3_ ceramics^34^,^35^,^36^,^37^,^38^. In addition, a small amount of watermarks labelled as ‘W’ (believed to correspond to 180°-domain boundaries)^28^,^34^, were often observed in coarse grained BaTiO_3_ ceramics and rarely appear in fine grains (Figs. 4b, 4f and 4h). This suggests the existence of 180° domains in coarse grained BaTiO_3_ ceramics after poling, indicating that either it was not possible to align all the 180° domains during poling or that the ceramics lost part of the domain alignment on removal of the field after the poling process.

**Figure 5:**
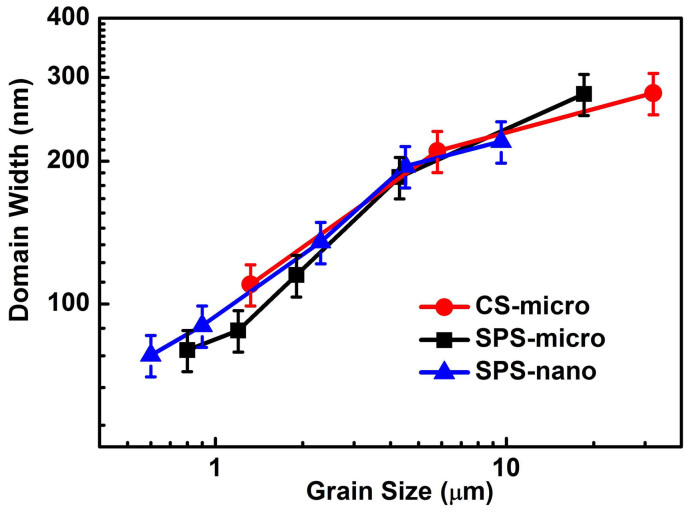
Grain size dependence of the average domain width for the CS and SPS BaTiO_3_ ceramics.

The domain width of the striped domain structure was measured at a large number of locations in ceramics with different grain size and an average was calculated. The resultant grain size dependence of the average domain width for BaTiO_3_ ceramics prepared by different sintering methods is shown in Fig. 5. It can be seen that the average domain width decreases with decreasing grain size for all BaTiO_3_ ceramics. From Fig. 5 it is clear that there is a deviation from the parabolic relationship between domain width and grain size previously reported in ferroelectric ceramics^36^,^39^. Deviations from the parabolic law were also found in other ferroelectric systems and have been recently discussed in the literature^40^,^41^. When the grain size is larger than 4 μm, the CS ceramics and SPS ceramics show a similar grain size dependence of the domain width. The value of the domain width of the fine-grained BaTiO_3_ ceramics is consistent with that typically reported in literature (~ 100 nm)^17^,^28^,^29^,^31^. The average domain width of the coarse-grained ceramics is much smaller than the previously reported values (larger than 500 nm)^17^,^34^.

## Discussion

It is well known that the dielectric and piezoelectric properties of ferroelectric ceramics include intrinsic and extrinsic contributions; the former originates from the deformation of the unit cell under an external electric or mechanical field, while the latter is mainly due to domain wall movement and point defects^42^,^43^,^44^,^45^,^46^,^47^,^48^. The domain wall contribution is determined by the domain wall density and domain wall mobility, which are also both influenced by many factors including grain size, back fields and defects^42^,^43^,^44^,^45^,^46^,^47^,^48^. The differences in the grain size dependence of the piezoelectric properties of the CS and SPS BaTiO_3_ ceramics can be interpreted based on the following aspects.

### Domain wall density

The average domain width decreases with the reduction of the average grain size in BaTiO_3_ ceramics sintered by both methods, as demonstrated in Fig. 5, which is in agreement with Refs. 38, 40. This means that the number of domain walls per volume (the domain wall density) increases with decreasing grain size. This could contribute to a maximum in domain wall activity, which would produce an enhancement of the dielectric and piezoelectric properties in correspondence with a specific grain size. Optimum domain wall density could be one possible reason for the maximum permittivity observed near 1 µm grain size in CS and SPS ceramics (see Fig. 1a), as also suggested in previous reports^13^,^15^,^16^,^17^,^18^,^19^,^21^. However, domain wall density is not the only factor which controls the domain wall contribution in ferroelectric/ferroelastic materials and additional factors should be taken into account in unravelling all the grain size effects observed.

### Back fields

Back fields are a result of restoring forces acting on domain walls during domain switching^49^. The back fields may oppose the switching during electric field loading, and assist the back-switching during electric field unloading. A larger grain boundary area in fine grained ceramics would produce a back field which would exert a clamping effect on domain walls making the ferroelectric/ferroelastic domains harder to switch during the application of an electric field^50^,^51^. This explains why the *E*_c_ value of the SPS ceramics decreases with increasing grain size (Fig. 3c). In the case of the SPS-nano ceramics, the piezoelectric constant and the remnant polarization both decrease with decreasing average grain size in the entire range studied (Figs. 1b, 3b). The decrease of *P*_r_ with decreasing grain size can be attributed to an increased effect of the back field in ceramics with smaller grains. Back fields can also induce a reduction of the *d*_33_ in accordance with the following scenarios, which may also overlap: a) the increased back field in ceramics with smaller grains might hinder domain alignments during DC poling; b) the back field reduces the alignment of the domains when the electric field is removed after the DC poling process.

### Point defects

**Figure 6:**
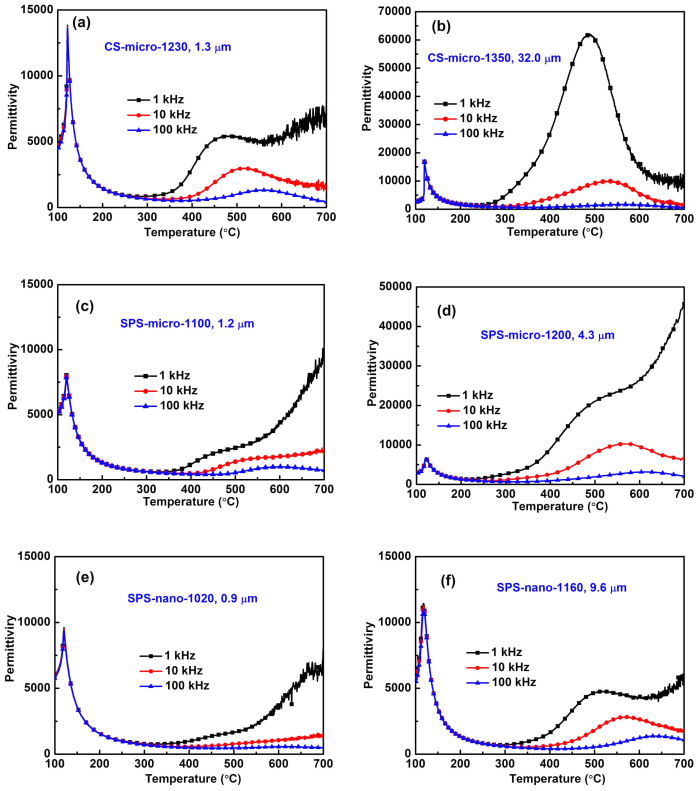
Temperature dependence of dielectric permittivity for BaTiO_3_ ceramics. (a), (b) CS-micro ceramics; (c), (d) SPS-micro ceramics; (e), (f) SPS-nano ceramics.

In order to achieve high density in conventional sintering, the sintering temperatures are higher and the dwelling times are much longer than those utilized in the SPS method. It was suggested that this represents a possible cause of point defects in the ceramics prepared by the conventional route^52^. Point defects tend to migrate to the domain boundaries or grain boundaries and subsequently pin the domain walls^53^,^54^,^55^,^56^,^57^. In our BaTiO_3_ samples, domain wall pinning effects seem to increase with increasing sintering temperature as demonstrated in Fig. 3 by the increase of *E*_c_ and the decrease of *P_max_* with increasing grain size in CS-micro ceramics. Figure 6 shows the temperature dependence of the dielectric permittivity of the BaTiO_3_ ceramics prepared from different powders using different sintering methods. The peaks near 120°C correspond to the Curie point *T*_c_ of BaTiO_3_. The broad peaks in the dielectric permittivity at high temperature from 400 to 700°C, often observed in perovskite-type ferroelectric oxides including BaTiO_3_, can be attributed to the motion of oxygen vacancies as suggested by the calculated activation energy reported in Ref. 58. The peak intensity increases with increasing sintering temperature for CS-micro ceramics (Figs. 6a and b). In addition, the X-ray Photoelectron Spectroscopy (XPS) analysis shows that the valence state of barium and oxygen changes with increasing sintering temperature, reflecting possible modifications in the Ba-O coordination and stoichiometry, which could be accommodated by the formation of oxygen vacancies (refer to supporting Figure S2). In Figs. 6c and d, the intensity of the high temperature dielectric peaks decreases with reduction of the sintering temperature for SPS-micro ceramics. Further evidence for the presence of point defects (i.e. oxygen vacancies) in SPS-micro ceramics sintered at high temperatures are represented by the existence of an additional current peak that appears in the current-electric field (I-E) curves at temperatures starting from 80°C (refer to supporting Figures S3a and S3b), an additional loss peak at a low frequency of about 100 Hz (refer to supporting Figures S3c and S3d) and by an asymmetric strain-electric field loop (refer to supporting Figures S3e and S3f). The high temperature broad peaks in the dielectric permittivity are almost absent in SPS-nano ceramics (Fig. 6e) due to the lower sintering temperature used.

The presence of point defects can reduce the domain wall mobility, thereby decreasing the extrinsic contribution of domain walls to the dielectric and piezoelectric properties. Domain wall pinning by point defects can have an important contribution to the reduction of the dielectric permittivity with increasing grain size in CS ceramics and for the lower permittivity of CS samples compared to SPS ceramics (Fig. 1a). For piezoelectric properties, the existence of point defects restricts domain switching under the DC poling process resulting in a poor domain alignment after poling, which leads to a lower piezoelectric constant. In addition, the small oscillating force used to measure the piezoelectric constant may not be large enough to counteract the pinning effect caused by the point defects resulting in the decrease of *d*_33_ with increasing grain size in the CS-micro ceramics. In CS-micro ceramics, the remnant polarization and the piezoelectric constant both decrease with increasing grain size over the entire range studied. This suggests that the effect of domain wall pinning increases with increasing sintering temperature, resulting in a larger coercive field and a significant decrease of *P*_max_ in CS-micro ceramics with the largest grain size (see Figs. 1b and 3a–3c). In Fig. 2, the coarse grained SPS ceramics exhibit more saturated P-E hysteresis loops and much larger *P*_r_ values than those of the CS-micro ceramics. In addition, the maximum polarization *P*_max_ of SPS ceramics increases with increasing grain size in the range studied (Fig. 3a). The effects of sintering temperature on domain wall activity is also evidenced by the different grain size dependence of the piezoelectric constant observed for the SPS-micro ceramics and SPS-nano ceramics (Fig. 1). The latter were sintered at much lower temperatures and show a constant increase in the *d*_33_ over the same grain size range. These observations indicate that when the sintering temperature becomes too high, the effect of domain wall pinning by point defects becomes the main factor in determining the observed grain size dependence of the piezoelectric and ferroelectric properties.

### Origin of the different grain size dependencies of the permittivity and piezoelectric constant

In order to clarify the different grain size dependencies of the permittivity and piezoelectric constant, the SPS-nano ceramics will be considered first as the contribution of point defects to the grain size dependence observed is presumably not dominant and the grain size dependence of the permittivity and *d*_33_ shows the most remarkable differences among the ceramics studied here (Figs. 1a, 1b). Considering the P-E loop (Fig. 2c) and the S-P loop (Fig. S4c) of the SPS-nano-1160, it can be inferred that in the region around the coercive field the domain switching process is dominated by 180° domain reorientation. This is evidenced by the steep change in polarization and the minor strain change in the region around the coercive field (P = 0), as shown in the hysteresis plots of Fig. 2c and Fig. S4c. In addition, the SEM images of the domain structure suggest an increased presence of 180° domains in SPS-nano-1160 compared to the other SPS-nano ceramics. Therefore, it is unlikely that the permittivity of SPS-nano-1160 is mainly dominated by the 180° domain wall contribution; otherwise it would not be the lowest among all the SPS-nano ceramics (Fig. 1a). On the other hand, the *d*_33_ of SPS-nano-1160 is the largest within the SPS-nano ceramics, due to the smaller back field experienced by this ceramic, which allowed for a higher degree of domain alignment during DC poling and a limited back-switching after poling. This is in agreement with the largest value of *P*_r_ in SPS-nano-1160, among all the SPS-nano ceramics studied. Since it was deduced that the contribution of 180° domain walls to the permittivity is not dominant, it can be inferred that the back field acts on 90° domains. By definition, the dielectric permittivity indicates the amount of polarization change that can be induced under the application of an electric field, thus it should be expected that the permittivity generally decreases with increasing domain alignment after DC poling, as confirmed by previous experiments^28^. Therefore the smallest permittivity in the SPS-nano-1160 with the largest grain size among the poled SPS-nano ceramics should be attributed to a smaller contribution of 90° domain walls due to a limited back-switching of 90° domains after poling, in comparison to the other SPS-nano ceramics (see also Supporting Information S4). The dominant contribution of 90° domain walls to the dielectric permittivity has been unambiguously proven in PbZr_0.2_Ti_0.8_O_3_ thin films^59^. With decreasing grain size, the *d*_33_ decreases because of the increase of the back field according to the scenarios a) and b) previously described, while the permittivity increases, because of a larger contribution of 90° domain walls. When the grain size becomes too small, the contribution of 90° domain walls decreases and the permittivity of the poled ceramics starts to decrease with decreasing grain size (below 1 µm in BaTiO_3_ ceramics). Similar arguments apply to the permittivity of the SPS-micro ceramics and the CS-micro ceramics, for which the grain size dependence of the *d*_33_ has already been explained.

### Summary of the observed grain size dependence

In the attempt to understand the grain size dependence observed in barium titanate ferroelectric ceramics, highly convoluted effects were observed on dielectric, piezoelectric and ferroelectric properties, which can be summarized as follows. The grain size dependence of the dielectric permittivity is mostly independent of both the starting powders and sintering process used. The maximum dielectric permittivity at a critical grain size of about 1 µm is mainly achieved by optimum density and mobility of 90° domain walls.

The grain size dependence of the piezoelectric constant is instead affected by additional factors related to ceramic processing, including particle size of the starting powder and sintering temperature. With increasing grain size, the *d*_33_ of CS-micro ceramics decreases over the whole grain size range; the *d*_33_ of SPS-micro ceramics first increases and then decreases with a peak at 4.3 μm grain size; the *d*_33_ of SPS-nano ceramics increases over the entire grain size range studied. The increase of *d*_33_ in the SPS-nano ceramics and in the SPS-micro ceramics below 4.3 μm is due to an increased domain alignment caused by a reduction of the back field exerted by grain boundaries with increasing grain size. Domain wall pinning by point defects is instead the main factor for the decrease of the *d*_33_ in SPS-micro ceramics above 4.3 μm and in CS-micro ceramics over the entire grain size range.

Regarding the ferroelectric properties, it was observed that the coercive field of SPS ceramics decreases with increasing grain size, while in CS ceramics it increases in ceramics with larger grains. The latter is attributed to an increased pinning effect on domain walls by point defects developed in ceramics sintered at high temperature. In ceramics where the point defects contribution is not dominant, the maximum and remnant polarization increase with increasing grain size.

## Conclusions

Grain size effects on dielectric, piezoelectric and ferroelectric properties of three different groups of BaTiO_3_ ceramics with distinct sintering temperature ranges were studied. This allowed for a wider view of the possible grain size dependencies which could be observed in ferroelectric ceramics. Grain size effect is influenced by contributions from ferroelectric domain walls, back fields and point defects developed during sintering at high temperatures. The maximum dielectric permittivity near 1 µm grain size is achieved by optimum density and mobility of 90° domain walls in all the BaTiO_3_ ceramics studied. In ceramics sintered by SPS at low temperatures (*T* < 1200°C), the grain size dependence of the piezoelectric and ferroelectric properties can be mainly attributed to the degree of domain alignment and the influence of the back field exerted by the grain boundaries. In ceramics sintered at high temperatures either by SPS or CS, there is an additional contribution from point defects, which increases with increasing sintering temperature and influences the domain wall contribution to the grain size effect observed. When studying grain size effects in ferroelectric materials, the point defects contribution should be minimized to avoid contradicting and misleading observations.

## Methods

### Powder preparation

Conventional micro-sized BaTiO_3_ powders were prepared from commercial BaCO_3_ (purity ≥ 99.0%) and TiO_2_ (purity ≥ 99.8%) powders. The BaCO_3_ and TiO_2_ powders were weighed according to the stoichiometric formula and ball-milled for 12 h in nylon pots with ZrO_2_ balls and alcohol as a milling media. The slurry was then dried and ground using an agate mortar and pestle. The powder mixture was calcined at 1050°C for 4 h. After the second ball-milling, a fine BaTiO_3_ powder with average particle size lower than 0.5 μm was obtained. Hydrothermal synthesized nano-powders of BaTiO_3_ with a particle size of 100 nm were also used as the precursor materials. The microstructures of different BaTiO_3_ powders are shown in Fig. S1.

### Sintering

Two kinds of sintering techniques were adopted to sinter BaTiO_3_ ceramics: conventional sintering (CS) and Spark Plasma Sintering (SPS). In the case of CS, the micro-sized BaTiO_3_ powders (Fig. S1a) were pressed into pellets of 15 mm diameter and 1 mm thickness, and then sintered at 1210°C, 1280°C and 1350°C for 2 h in air (referred as CS-micro-1210, CS-micro-1280, and CS-micro-1350 for abbreviation). In the case of the SPS samples, BaTiO_3_ micro-sized powders were sintered in vacuum for 5 minutes under an uniaxial pressure of 85 MPa at 1080°C, 1100°C, 1120°C, 1200°C and 1240°C (referred as SPS-micro-1080, SPS-micro-1100, SPS-micro-1120, SPS-micro-1200 and SPS-micro-1240 for abbreviation) using a SPS furnace (HPD-25/1 FCT systeme GmbH). The 100 nm nanopowder (Fig. S1b) was sintered by the SPS unit at 1000°C, 1020°C, 1040°C, 1060°C and 1160°C (referred as SPS-nano-1000, SPS-nano-1020, SPS-nano-1040, SPS-nano-1060 and SPS-nano-1160 for abbreviation). All the SPS samples were annealed in air at 900°C for 2 h to eliminate the presence of any residual carbon and reduction effects incurred during SPS process.

### Characterization

The density of the ceramics was measured by the Archimedes method. For electrical characterization, disk-shaped specimens were coated with a silver paint (Gwent Electronic Materials Ltd, C2011004D5, Pontypool, UK) on the top and bottom surfaces and fired at 600°C for 20 min. Poling was carried out at 105°C in silicon oil under an electric field of 3.0 kV/mm for 30 min. The piezoelectric *d*_33_ coefficient was measured using a Berlincourt-type *d*_33_ meter (model YE 2730A, Sinocera Piezotronics, China). The dielectric properties were measured using an impedance analyzer (Agilent 4294A). The polarization hysteresis (P-E) loops were traced using a ferroelectric hysteresis measurement tester (NPL, Teddingdon, UK)^60^,^61^. For the microstructure and domain configuration characterization, the poled specimens were mirror-polished and chemically etched for 10 s in an aqueous solution of 5% HCl with a small amount of HF (3 drops of HF:20 ml HCl solution). The microstructure and domain structure observations were performed using a scanning electron microscope (SEM; model JEOL JSM 6300). X-ray photoelectron spectroscopy (XPS; ESCALAB MK II, VG Scientific) was carried out to study the valence state of ions in the ceramics sintered at different temperatures.

## Author Contributions

T.Y.Q., Z.J.L., K.V., W.C.L., R.M., V.G. and Y.H.X. wrote the main manuscript text, W.Y.Q. prepared Figure 1, T.Y.Q. prepared Figures 2–6 and Figures S1, 3, S.B.G. and Y.H.T prepared Figures S2. All authors reviewed the manuscript.

## Supplementary Material

Supplementary Information Unfolding grain size effects in Barium Titanate ferroelectric ceramics

## Figures and Tables

**Table 1:** Dielectric and piezoelectric properties of the poled BaTiO_3_ ceramics prepared from micro-sized and nano-sized powders at room temperature. The values of the relative density, grain size, piezoelectric constant *d_33_*, orthorhombic-tetragonal transition temperature *T*_O-T_ and Curie point *T*_C_ data are included

Sintering Condition	Relative Density (%)	Average Grain Size (μm)	Permittivity at 1 kHz	tanδ (%)	*d*_33_ (pC/N)	*T*_C_ (°C)	*T*_O-T_ (°C)
*CS-micro-1230*	95.2	1.3	3220	2.30	410	120.2	28.1
*CS-micro-1280*	97.8	5.8	2970	2.17	300	121.5	25.7
*CS-micro-1350*	98.6	32.0	2730	2.44	180	122.3	23.2
*SPS-micro-1080*	98.5	0.8	4010	2.58	311	121.5	30.7
*SPS-micro-1100*	99.1	1.2	4450	2.60	339	121.2	30.2
*SPS-micro-1120*	99.0	1.9	3670	2.41	343	121.5	30.7
*SPS-micro-1200*	99.3	4.3	3400	2.35	432	123.6	26.5
*SPS-micro-1240*	98.9	18.5	2900	2.68	389	124.1	21.6
*SPS-nano-1000*	98.2	0.6	5340	1.32	330	120.8	30.2
*SPS-nano-1020*	98.5	0.9	5800	1.17	340	120.6	29.6
*SPS-nano-1040*	99.3	2.3	5080	1.19	375	123.8	24.9
*SPS-nano-1060*	99.2	4.5	4500	1.11	420	123.9	21.5
*SPS-nano-1160*	99.5	9.6	3960	1.38	425	125.5	18.1
