# Supplementary material for: Unfolding grain size effects in barium titanate ferroelectric ceramics
Source: Sci Rep. 2015 May 7;5:9953. doi: 10.1038/srep09953 (PMC4423446; doi:10.1038/srep09953)
Supplement: Supplementary Information — Unfolding grain size effects in Barium Titanate ferroelectric ceramics [file srep09953-s1.doc]

**Supporting Information**

Unfolding grain size effects in Barium Titanate ferroelectric ceramics

Yongqiang Tan,^1,4^ Jialiang Zhang,^1^* Yanqing Wu,^1^ Chunlei Wang,^1^ Vladimir Koval,^2^ Baogui Shi,^3^ Haitao Ye,^3^ Ruth McKinnon,^4^ Giuseppe Viola^4,5^ & Haixue Yan^4^

^1^School of physics, State Key Laboratory of Crystal Materials, Shandong University, Jinan 250100, P. R. China

E-mail: [*zhangjialiang@sdu.edu.cn*](mailto:zhangjialiang@sdu.edu.cn)

^2^Institute of Materials Research, Slovak Academy of Sciences, Watsonova 47, 040 01 Kosice, Slovakia

^3^School of Engineering and Applied Science, Aston University, Birmingham B4 7ET, United Kingdom

^4^School of Engineering and Materials Science, Queen Mary University of London, Mile End Road, London , E1 4NS, United Kingdom

^5^Department of Applied Science and Technology, Institute of Materials Physics and Engineering, Corso Duca degli Abruzzi 24, 10129 Torino, Italy

**Figure S1** shows the microstructure of both the micro-sized (Fig. S1a) and nano-sized (Fig. S1b) BaTiO_3_ powders used in this study. It can be seen that the powders show a uniform particle size distribution. For conventional powders obtained through solid state reaction and ball-milling, the average particle size is about 0.5 μm. Nano-sized powders prepared by the hydrothermal method consist of particles of about 100 nm.

**Figure S2** shows the X-Ray Photoelectron Spectroscopy (XPS) spectra of barium 3d, titanium 2p and oxygen 1s for the ceramics sintered by SPS at different temperatures. The present data show that the spectrum relative to titanium does not show any great differences. However, the XPS spectrum of barium shows remarkable changes. Considering the curve relative to the ceramic sintered at 1100°C it can be seen that there is a shoulder on the left side of the two main peaks in the barium spectra (Fig.S2a) and a shoulder on the left side of the main peak in the oxygen spectra (Fig.S2c). The intensity of these shoulders increases as the sintering temperature increases in both the spectra. At the same time the intensity of the original main peaks decreases in both the spectra. In the ceramics sintered at 1240°C the original main peaks almost disappear in the barium spectra (Fig.S2a), and the intensity of the original main peak of the oxygen spectra significantly reduces (Fig.S2c). This indicates the increase of contribution from oxygen vacancies with increasing sintering temperatures.

**Figure S3** shows the typical differences observed in the current-electric field curves (Figs. S3a and S3b), dielectric properties (Figs. S3c and S3d) and strain-electric field loops (Figs. S3e and S3f) for the ceramics sintered by SPS at 1100 °C (S3a, S3c and S3e) and 1240 °C (S3b, S3d, and S3f) using the micro-sized powder. It can be noticed that the ceramics sintered at 1240 °C show an additional small current peak close to the switching peak in the negative electric field range (Fig. S3b). This additional peak becomes visible because the main current peaks corresponding to domain switching shifts towards the negative electric field side (Fig.S3b). This suggests the existence of a positive internal bias possibly related to the presence of point defects. The presence of defect species is also evidenced by an additional peak found in the imaginary part of the permittivity of a virgin unpoled sample in the low frequency range at about 100 Hz (Fig. S3d). The presence of a positive internal bias is also supported by the asymmetry in the strain-electric field loop (S-E) which shows a much smaller strain in the negative electric field range in Fig. 3f.

**Figure S4** shows the current-electric field (I-E) loops (Fig.S4(a)), the strain-electric field (S-E) loops (Fig.S4(b)), the strain-polarization (S-P) loops (Fig.S4(c)), and the strain rate-electric field (S_R_-E) loops (Fig.S4(d)) of the ceramics prepared from nano-sized powder using SPS at 1000°C (0.6 μm) and 1160°C (9.6 μm) at regime conditions.^[1]^ It can be seen that the current peaks corresponding to domain switching in the former ceramics are much broader than those of the ceramics sintered at 1160°C, indicating that polarization reversal occurs in a broader electric field range. In addition, it can be observed that larger strain amplitude (difference between maximum and minimum strain in the S-E loop) and smaller polarization is achieved in the S-E loop of smaller grain size ceramics (Fig.S4(c)). This suggests that in ceramics with small grains the contribution from 90° domain switching is predominant, while the larger grain size ceramics have an increased contribution from 180° domain wall movement which determines the observed higher polarization and the larger hysteresis in the S-P loops (reflecting a lag between polarization and strain ^[2]^) as shown in Fig.6(c). This is also further confirmed by the much smaller strain change with respect to the polarization change in the region around P=0 (see Fig.6c). Figure 6(d) shows the strain rate as a function of the electric field ^[2]^, where the peaks during loading have been indicated with ±E_F_ and the peaks during unloading/reversal are labeled with ±E_B_. At the moment it is still not clear if these peaks can only be associated with the non-180° domain switching/back switching, or they also include the contribution of reversible electric field induced transitions according to the framework proposed in Ref. 3. In the latter, the in-situ X-Ray experiments demonstrated that the BaTiO_3_ ceramics experience reversible electric field-induced transitions, which represents an additional contribution to the strain together with the piezoelectric effect and 90° domain wall movement. It is possible that the peaks observed in the S_R_-E plot represent the characteristic fields that may trigger the forward transition during electrical loading (±E_F_) and the backward transition during electric field removal/reversal (±E_B_). It can be seen that ceramics with larger grains sintered at 1160°C (SPS-1160) shows lower ±E_F_ and lower ±E_B_ (Fig.S4(c)). In addition, in the ceramics sintered at 1160°C the strain rate peaks are sharper and in the high field range the strain rate tends to a plateau value suggesting that the process (either phase transition or domain switching or a convolution of both) is complete and that 90° domain switching is saturated, which is not the case for the ceramics sintered at 1000°C. Furthermore, the ceramics with larger grains show higher strain rate in the low electric field range and smaller strain rate in the high electric field range in comparison to the smaller grained ceramics. In ceramics sintered at 1000°C the strain rate peaks at (±E_B_) are found during the electric field unloading, while in ceramics sintered at 1160°C the peaks at (±E_B_) appear when reversing the electric field sign at very low electric field values, suggesting a polarization reversal process which contains two steps of 90° domain switching as found in PZT.^[2]^ All these observations indicate that the electric field-induced forward process is facilitated in larger grained ceramics, while that the back switching of 90° domains is retarded in ceramics with larger grains. This further justifies the interpretation of the lower permittivity in SPS nano-ceramics with larger grains given in the paper.

1. Tan, Y. Q. Zhang, J. L. Wang, C. L. Viola, G. & Yan, H. X. Enhancement of electric field-induced strain in BaTiO_3_ ceramics through grain size optimization. *Phys. Status Solidi A*, 1–6 (2014) (DOI 10.1002/pssa.201431233).

2. Viola, G. *et al*. Contribution of piezoelectric effect, electrostriction and ferroelectric/ferroelastic switching to strain-electric field response of dielectrics. *J. Adv. Dielectr.* **3**, 1350007 (2013).

3. Ghosh, D. *et al*. Domain wall displacement is the origin of superior permittivity and piezoelectricity in BaTiO_3_ at Intermediate grain sizes. *Adv. Func. Mater*. **24**, 885–896 (2014).

**Figure S1.** SEM images of (a) micro-sized and (b) 100 nm barium titanate powders.


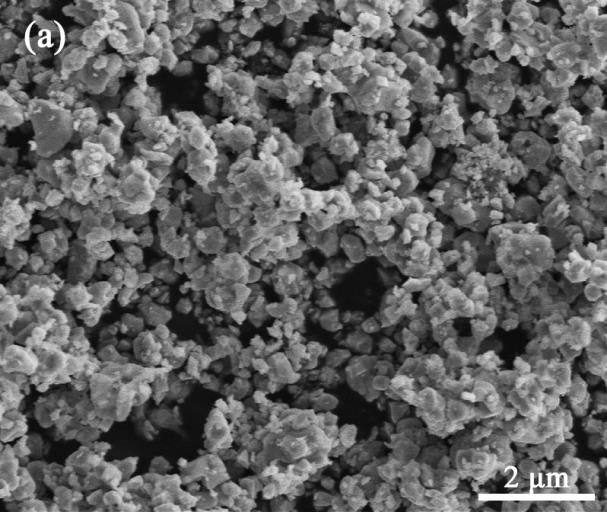

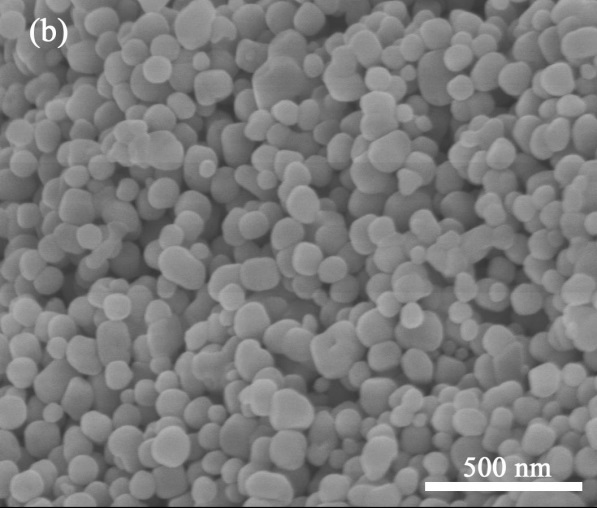


**Figure S2.** X-ray Photoelectron Spectroscopy of BaTiO_3_ ceramics conventionally sintered at different temperature using micro-sized powder. a) Barium 3d, b) Titanium 2p and c) Oxygen 1s spectra

**Figure S3.** Current-electric field loops measured at different temperatures in the range 80-150 °C for the ceramics prepared by the SPS technique from micro-sized powders at (a) 1100 °C and (b) 1240 °C. The frequency dependence of the real and imaginary parts of the dielectric permittivity at room temperature for the ceramics sintered at (c) 1100 °C and (d) 1240 °C. The bipolar strain-electric field loops measured at room temperature for the ceramics sintered at (e) 1100 °C and (f) 1240 °C.

**Figure S4.** (a) Current- electric field (I-E) loops, (b) the strain-electric field (S-E) loops, (c) the strain-polarization (S-P) loops, and (d) the strain rate-electric field (S_R_-E) loops of the ceramics prepared from nano-sized powder using SPS at 1000°C and 1160°C.
